# Crosslinking and mass spectrometry suggest that the isolated NTD domain dimer of Moloney murine leukemia virus integrase adopts a parallel arrangement in solution

**DOI:** 10.1186/1472-6807-13-14

**Published:** 2013-07-11

**Authors:** Daniel R Henriquez, Caifeng Zhao, Haiyan Zheng, José J Arbildua, Mónica L Acevedo, Monica J Roth, Oscar Leon

**Affiliations:** 1 Programa de Virologia ICBM, Facultad de Medicina, Universidad de Chile, Independencia 1027, Santiago, Chile; 2 Mass spectrometry facility at CABM, UMDNJ, New Jersey, USA; 3 Present address: Facultad de Ingeniería y Ciencias, Universidad Adolfo Ibañez, Santiago, Chile; 4 Department of Biochemistry, RWJMS-UMDNJ, Piscataway, New Jersey, USA

## Abstract

**Background:**

Retroviral integrases (INs) catalyze the integration of viral DNA in the chromosomal DNA of the infected cell. This reaction requires the multimerization of IN to coordinate a nucleophilic attack of the 3’ ends of viral DNA at two staggered phosphodiester bonds on the recipient DNA. Several models indicate that a tetramer of IN would be required for two-end concerted integration. Complementation assays have shown that the N-terminal domain (NTD) of integrase is essential for concerted integration, contributing to the formation of a multimer through protein-protein interaction. The isolated NTD of Mo-MLV integrase behave as a dimer in solution however the structure of the dimer in solution is not known.

**Results:**

In this work, crosslinking and mass spectrometry were used to identify regions involved in the dimerization of the isolated Mo-MLV NTD. The distances between the crosslinked lysines within the monomer are in agreement with the structure of the NTD monomer found in 3NNQ. The intermolecular crosslinked peptides corresponding to Lys 20-Lys 31, Lys 24-Lys 24 and Lys 68-Lys 88 were identified. The 3D coordinates of 3NNQ were used to derive a theoretical structure of the NTD dimer with the suite 3D-Dock, based on shape and electrostatics complementarity, and filtered with the distance restraints determined in the crosslinking experiments.

**Conclusions:**

The crosslinking results are consistent with the monomeric structure of NTD in 3NNQ, but for the dimer, in our model both polypeptides are oriented in parallel with each other and the contacting areas between the monomers would involve the interactions between helices 1 and helices 3 and 4.

## Background

Protein-protein interactions play a fundamental role on the assembly of multimeric complexes of IN to carry out two-end concerted DNA integration [1-3]. Retroviral integrase structures are organized in three domains, a central domain that contain the catalytic site (CCD), a N-terminal domain (NTD) that binds zinc and a C-terminal domain (CTD) having a SH3 fold [4].

Biochemical and genetic studies have shown that the NTD of integrase (IN) is involved in protein-protein interactions favoring protein multimerization and stabilization of the DNA-IN complex [5-8]. *Prototype foamy virus* (PFV) structure shows binding of this domain to LTR [9]. A polypeptide containing the first 105 amino acids of Mo-MLV IN, expressed in *E*. *coli*, is functionally active since it complements *in trans* an IN mutant lacking the NTD. Gel filtration chromatography indicates that this NTD behaves as a dimer in solution [8]. Similar observations have been made in HIV-1 IN [6]. The 3D structure of HIV-1 IN NTD, solved by NMR showed the dimer interface is highly hydrophobic and it includes the α-helices 3 and 4, and the N-terminal of helix 1 [10].

The NTD is essential for 3’ processing and strand transfer, however determining its role in the integration process in lentiviruses and oncogenic viruses has been difficult due to the absence of the full-length structure of IN and the complexity of the protein-protein and protein-DNA interactions involved in the synaptic complex. Several models based on the partial 3D structure of IN fragments have been proposed for HIV-1 IN and ASV [11-13]. The X-ray structure of a tetrameric integrase complex of the PFV IN and processed U5 DNA was solved [14-16]. In this complex, the NTDs of the external subunits have been located at distal positions of the complex [17], however their structure was unresolved in the crystal structure. The quaternary structure of HIV integrase in solution has been examined by small and wide X-ray angle scattering and chemical crosslinking [18]. It has been concluded that integrase can assemble as a tetramer by the interaction of two different dimers: one of them is stabilized by interactions between the CCDs of two subunits while the other dimer is stabilized by interactions of one NTD with the CCD, CTD, and NTD of the other subunit. The interaction between the NTDs in the latter dimer was detected by chemical crosslinking [18].

**Figure 1:**
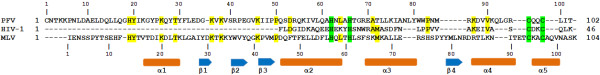
**Sequence alignment of PFV, HIV-1 and Mo-MLV NTD-IN.** The secondary structure of Mo-MLV is shown below. α-helices are marked as orange cylinders, and β-strands by blue arrows. Yellow boxes: at least two identical residues, Green boxes: three identical residues. Accession codes: PFV IN, CAA68999; HIV-1 IN, AAC83550 and Mo-MLV IN, P03355.

A sequence alignment of the NTD of Mo-MLV, PFV and HIV-1 integrases is presented in Figure 1. The NTD of Mo-MLV IN is 45 amino acids larger than the NTD of HIV-1 IN [8]. PFV IN also contains an extra region of 50 amino acids before the zinc binding domain. Since no quaternary structural information for Mo-MLV IN is available, in this work we explored the use of cross-linking in order to identify lysine residues that are within the range of the cross-linking spacer within the monomer or the dimer on the NTD. Cross-linked peptides were identified and sequenced by MALDI-TOF MS/MS spectroscopy. Based on these results and the 3D coordinates available in 3NNQ, a model of the NTD dimer was built. This model suggests a parallel arrangement of the NTDs.

## Results and discussion

The NTD of Mo-MLV integrase behaves as a dimer in solution according to gel filtration on Superose 12 and favors multimerization of IN [6,8]. In complementation studies it has been shown that addition of an isolated NTD domain to an IN construct lacking this domain restores the activities of IN. However, there is no information on the structure of the dimer of Mo-MLV in solution and how it interacts with the other IN domains. In this work we carried out chemical crosslinking to identify lysine residues that were located on the interface of the dimer in order to determine the regions involved in dimerization. This procedure involved the analysis of the crosslinked peptides on the isolated dimer and uncrosslinked monomers by mass spectrometry [19-21].

### Chemical cross-linking of IN 1–105 with BS^3^

**Figure 2:**
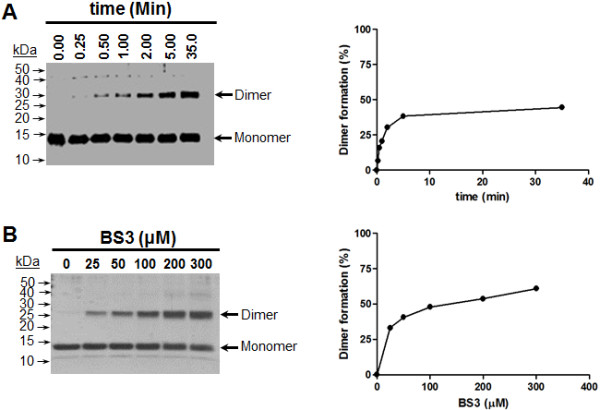
**Chemical cross-linking of IN1-105.** The reaction was carried out in 50 mM Hepes pH 7.8. IN 1 –105 and BS^3^ was incubated at 25°C in 40 μL at the times and concentrations indicated. The reaction was stopped with 50 mM lysine pH 8.0. **(A)** Crosslinking kinetics. 56 μM of IN 1–105 were incubated with 100 μM of BS^3^, aliquots were removed at the times indicated. *Left*, SDS-PAGE (15% acrylamide) silver stained (4 μg of protein were loaded in each lane). *Right*, formation of crosslinked IN 1–105 dimer (percent); quantification of dimeric and monomeric band were carried out with the imageJ program. **(B)** Effect of BS^3^ in crosslinking. 56 μM IN 1–105 and BS^3^ (0 to 300 μM) were incubated for 35 min at 25°C, aliquots containing 4 μg were loaded on a 12% acrylamide gel. *Left*, SDS-PAGE (12%) Coomasie brilliant blue stained gel. *Righ*t, formation of crosslinked IN 1–105 dimer.

Wild type IN 1–105 and K104C IN 1–105 were expressed in *E*. *coli* and purified by chromatography on Ni-NTA agarose. The proteins were 95% pure as judged by SDS-PAGE (result not shown). Wild type IN 1–105 was crosslinked with bis[sulfosuccinimidyl] suberate (BS^3^) in 50 mM Hepes pH 7.8 at 25°C. Figure 2A shows the results of the reaction kinetics using 56 μM of IN 1–105 and 100 μM BS^3^. A protein band migrating near 29 kDa corresponds to the expected dimer. In order to determine the effect of BS^3^ on IN 1–105 crosslinking, BS^3^ (between 0 and 300 μM) was used. The reactions were carried out at 25°C for 35 min and the results are shown in Figure 2B. Above 200 μM of BS^3^, other high molecular weight products besides the dimer are observed. 100 μM BS^3^ was used for crosslinking in the following experiments. Under these conditions approximately 50% of the protein was crosslinked.

**Figure 3:**
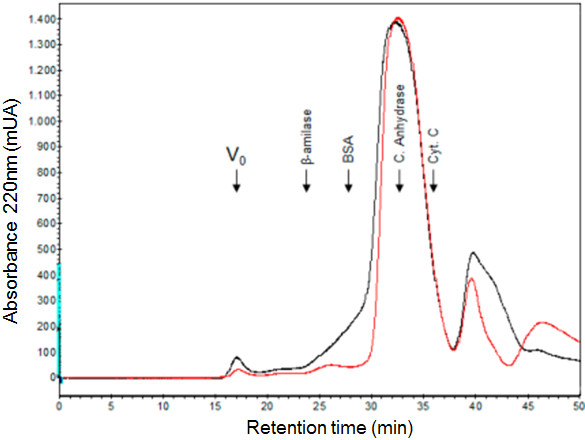
**Characterization of crosslinked product for molecular exclusion.** 2 mg of IN 1–105 were crosslinked in 200 μL and loaded on a Superdex S-200 column equilibrated in 10 mM tris pH 7.5, 0.5 M NaCl, 1 mM DTT and 5% glycerol at a flow rate of 0.5 mL/min. 0.5 mL of each fraction were collected. Protein elution was monitored by the absorbance at 220 nm. The chromatographic profile of the crosslinked protein is shown in black, and unmodified control protein is shown in red. The elution position of molecular weight markers is indicated by arrows. The markers used were: β-amylase (200 kDa), serum albumin (66 kDa), carbonic anhydrase (29 kDa) and cytocrome C (12.4 kDa). The (V_0_) indicated the void volume (dextran blue).

In order to determine the size of the crosslinked protein complex, the crosslinking mixture was analyzed by gel filtration on Superdex S-200 under nondenaturing conditions. The elution profiles of crosslinked and unmodified IN 1–105 were almost identical (Figure 3), and the majority of the crosslinked IN 1–105 (~95%) elutes at the same position of the unmodified dimer. This result indicates that, under the conditions of the reaction, intermolecular crosslinking occurs within each dimer and rules out crosslinking between the dimers.

### Identification of intramolecular and intermolecular crosslinking peptide with BS^3^

**Figure 4:**
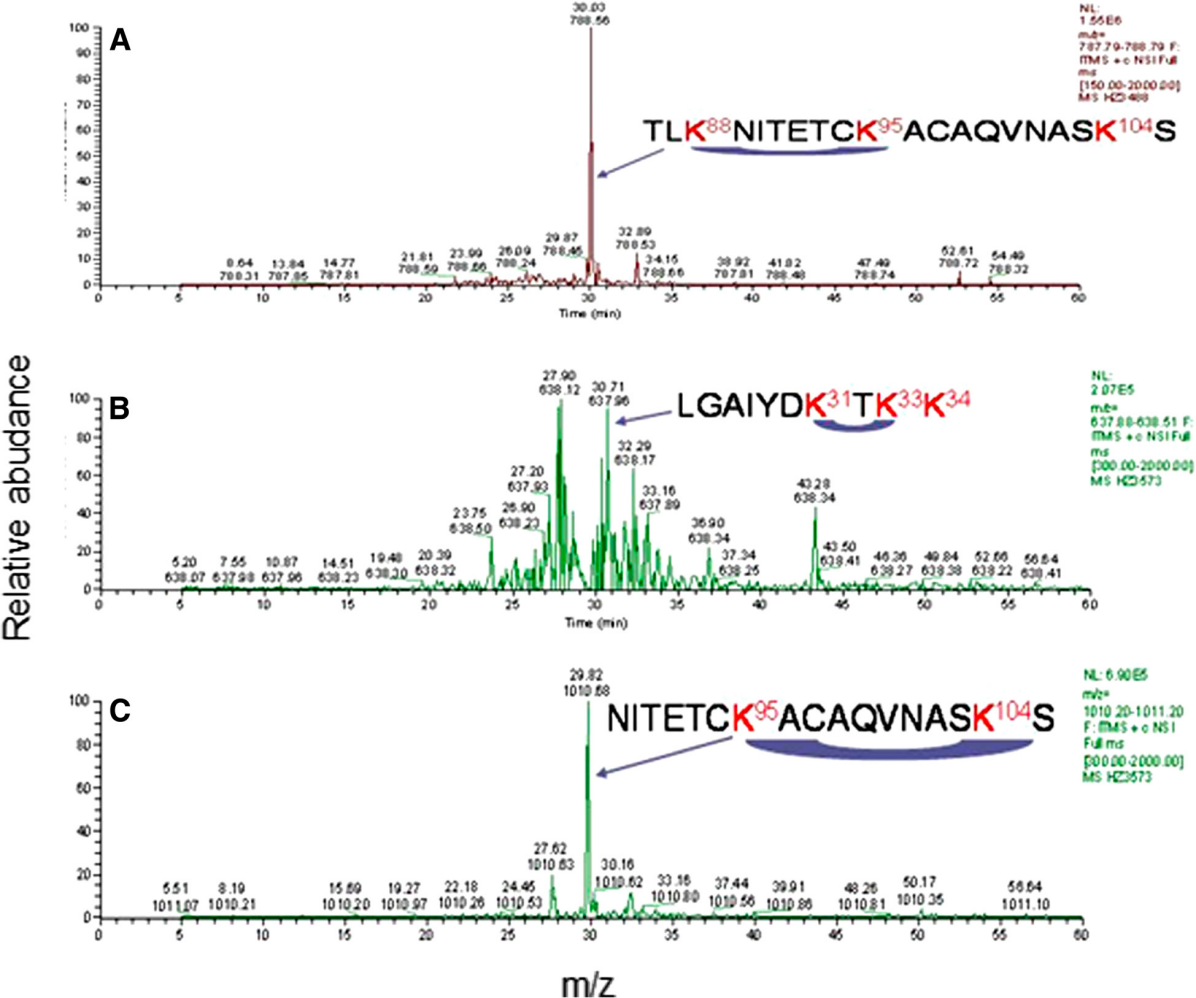
**LC-MS/MS peptide map profile of N-terminal domain of Mo-MLV integrase monomer crosslinked gel band digested with trypsin. (A)**, in the sample the peak corresponds to the peptide 86–105 with two cross-linked lysines (K88 and K95). **(B)**, the peak corresponds at the peptide 25–34 with intramolecular cross-linked between K31 and K33, **(C)** in the monomeric sample the peak corresponds at the peptide 89–105 with intramolecular cross-linked between K95 and K104.

**Figure 5:**
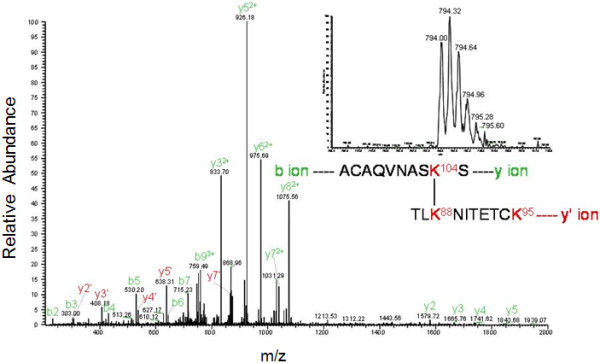
**LC-MS/MS peptide map profile of N-terminal domain of Mo-MLV integrase monomer crosslinked gel band.** Samples were digested overnight at 37°C with 0.8 μg of trypsin. The image shows the ionization and sequence of the LC peak (indicated in the inset) corresponding at the intramolecular peptide 96–105 and 86–95 with K88 and K104 modified.

Chemical crosslinking combined with mass spectrometry (MS) provides a useful method for inferring sites of protein-protein interactions and for mapping the topology of protein complexes. We used the bi-functional crosslinking reagent BS^3^ that reacts with primary amine groups of lysines and the NTD integrase of Mo-MLV. In these experiments IN 1–105 was crosslinked with BS^3^ and the dimeric and monomeric band was separated by SDS-PAGE, subjected to digestion with trypsin or chymotrypsin and analyzed by LC-MS/MS as indicated in Materials and Methods, being compared with the MS/MS profile of the unmodified IN 1–105, sequencing only differential peaks (Tables 1 and 2). In these cases only two informative sequences were used and classified as (1) *intramolecular crosslinked peptides* (*or looplinks*) *and* (**2**) *intermolecular crosslinked peptides*. Information about intramolecular crosslinking was obtained from reaction products in which both functional groups of the crosslinker reacted with lysine residues that reside in the same polypeptide chain (see Figures 4 and 5, Table 1). These peptides were sequenced by MS/MS and corresponded to the intramolecular crosslinking of: (1) residues K88 and K95 in (TLK^88^NITETCK^95^ACAQVNASKS), (2) residues K31 and K33 in LGAYDK^31^TK^33^K, (3) residues K95 and K104 in NITETCK^95^ACAQVNASK^104^S, (4) and residues K88 and K104 in TLK^88^NITETCKACAQVNASK^104^S. The informative contact position of lysines identified with the looplinks (see Table 1), was used to analyze the 3D crystal structure of the NTD of Mo-MLV integrase (3NNQ) in PDB data bank. We used this structure as a template and examined the distances between the crosslinked lysines. A maximum limit for productive crosslinking of 21.3 Å has been used as a restraint for the distance between the Cβ of two crosslinked lysines using BS^3^[22]. The distances between the crosslinked lysines were within the 21.3 Å limit, although the crosslinked K88-K104 was near this limit. Thus, our results are in agreement with the monomeric structure of the NTD domain in 3NNQ (Table 1).

**Table 1:** Looplinked peptides sequences of BS^3^ crosslinked peptides of Mo-MLV IN NTD and the Cβ distances between modified lysine residues in the generated model

**Crosslinking**	**Peptide 1**	**Lysine involved**	**Distance (Å)**
Looplinks	TLK^88^NITETCK^95^ACAQVNASKS	88 – 95	15.45
	LGAYDK^31^TK^33^K	31 – 33	7.32
	NITETCK^95^ACAQVNASK^104^S	95 – 104	16.15
	TLK^88^NITETCK - ACAQVNASK^104^S	88 – 104	20.74

The distances were defined using SwissPdbViewer program.

**Table 2:** Intermolecular peptides sequences of BS^3^ crosslinked peptides of Mo-MLV IN NTD and the Cβ distances between modified lysine residues (bold type) in the dimeric model

**Crosslinking**	**Peptide 1**	**Peptide 2**	**Lysine involved**	**Distance (Å)**
Intermolecular crosslinking	TVTDIKDLTK^24^LGAIY	TK^24^L	24 – 24 *	15.14
	LGAYDK^31^TK	MIENSSPYTSEHFHYTVTDIK^20^DLTK^24^LGAIYDK	31 – 20/24	20.02
	DK^31^TKKY	TVTDIK^20^DLTKL	31 – 20 *	20.02
	MK^68^ALLER	TLK^88^NITETCK	68 – 88	13.57

()* Peptides obtained by chymotrypsin digest.

The distances were defined using SwissPdbViewer program.

**Figure 6:**
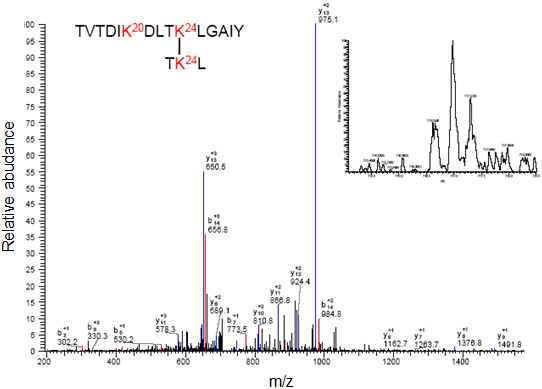
**LC-MS/MS profile of N-terminal domain of Mo-MLV integrase cross-linked with BS^3^.** Samples in solution were digested overnight at 37°C with chymotrypsin in a ratio of 1:20 wt/wt (enzyme: protein). The image shows the ionization and sequence of the LC peak (indicated with asterisk in the inset) corresponding at the intermolecular peptide 15–29 and 23–25 with K24 and K24 crosslinked.

**Figure 7:**
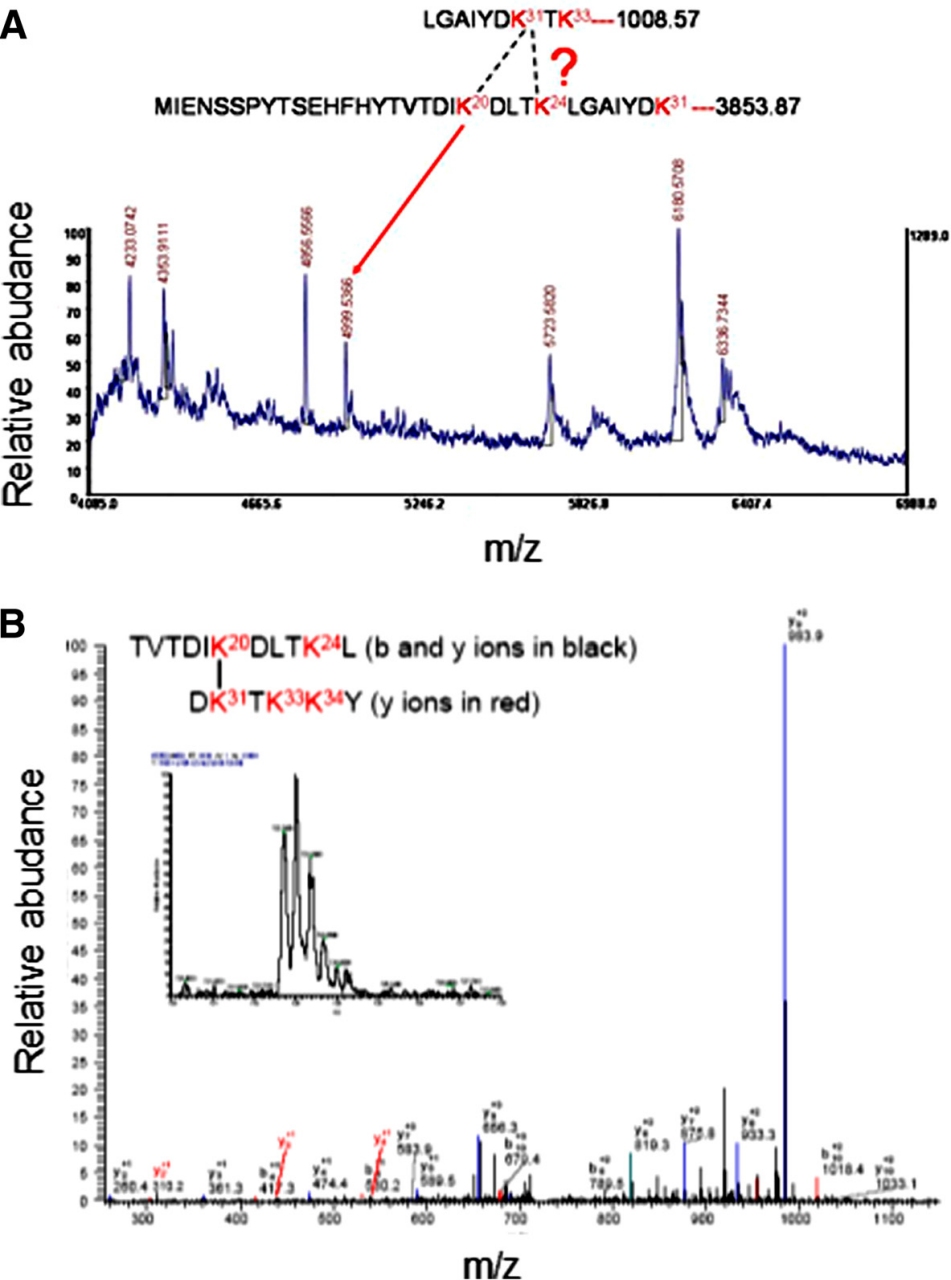
**Intramolecular crosslinking peptide. (A)** MALDI-TOF profile of the tryptic digest of dimeric band of IN 1–105 crosslinked with BS^3^. Samples were digested overnight at 37°C with trypsin in a ratio of 1:20 wt/wt (enzyme: protein). The image showed the peak 4999.5366 (m/z) corresponding at the intermolecular peptide 25–33 and 1–31 with K31 and K20 or K24 modified. **(B)** LC-MS/MS profile of the chymotryptic IN 1–105 were cross-linked with BS^3^. Samples were digested overnight at 37°C with chymotrypsin in a ratio of 1:20 wt/wt (enzyme:protein). The image shows the ionization and sequence of the LC peak (indicated in the inset) corresponding at the intermolecular peptide 15–25 and 30–35 with K31 and K20 crosslinked.

**Figure 8:**
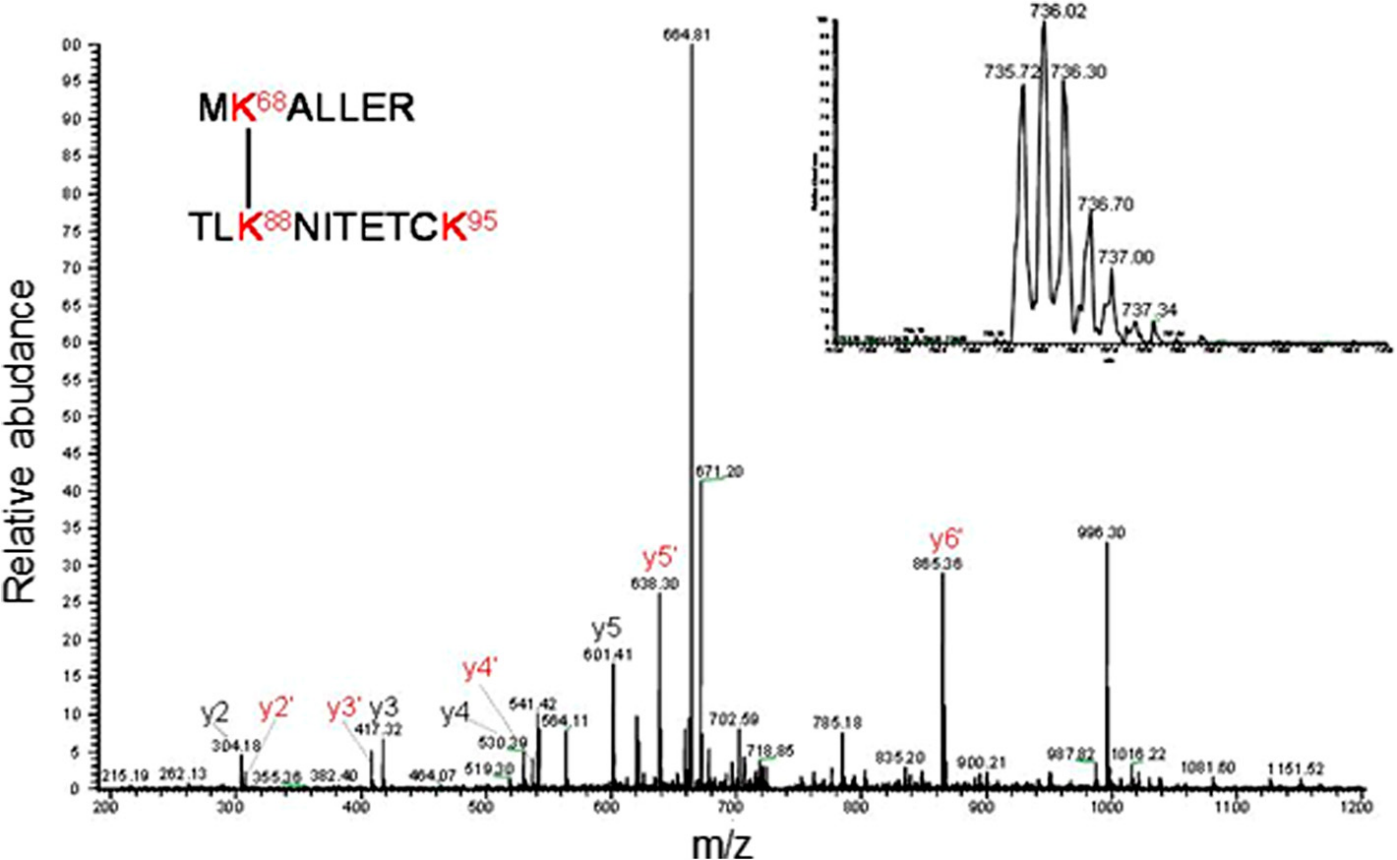
**Intermolecular crosslinking.** LC-MS/MS peptide map profile obtained of the dimer crosslinked gel band of N-terminal domain of Mo-MLV integrase. The dimer was digested with 0.8 μg of trypsin at 37°C overnight. The image shows the ionization and sequence of the LC peak (indicated in the inset) corresponding at the intermolecular peptide 67–73 and 86–95 with K68 and K88 crosslinked with BS^3^.

The next step was to determine the contact zones of the dimeric protein-protein interface, using the information of the reaction products in which the crosslinked functional groups belonged to different polypeptides (see Table 2). Figure 6 shows the profile of one of these differential peptides obtained after digestion of the dimeric band with chymotrypsin. The peptide was sequenced yielding the sequence TVTDIKDLTK^24^LGAIY – TK^24^L, which contain the crosslinked residues K24 and K24 suggesting the proximity between both N-terminal ends of the polypeptides. When a similar analysis of the crosslinked protein was carried out using trypsin, a large peptide (m/z nearly 5,000) was observed (Figure 7A) that would correspond to crosslinking between K31 and either K20 or K24 indicated for the mass of the fragment (bioworks software analysis) of 4999.5366 m/z. This peptide could not be sequenced by MS/MS due to its large size. However when the crosslinked IN 1–105 dimer was digested with chymotrypsin, a peptide corresponding to crosslinking of residues K20 and K31 on each of the polypeptide chains was identified and sequenced (Figure 7B). These results indicate that crosslinking occurs between K20 and K31 of different polypeptides. A third crosslinked peptide was identified after tryptic digestion of the crosslinked dimer in the MS/MS sequences as MK^68^ALLER-TLK^88^NITETCK indicating that K68 and K88 are crosslinked (Figure 8). The intermolecular crosslinking pattern identified shows a particular distribution along the NTD (see Table 2). These results allow us to produce a 3D model and characterize the dimeric interface.

### Model of the NTD dimer of Mo-MLV integrase

**Figure 9:**
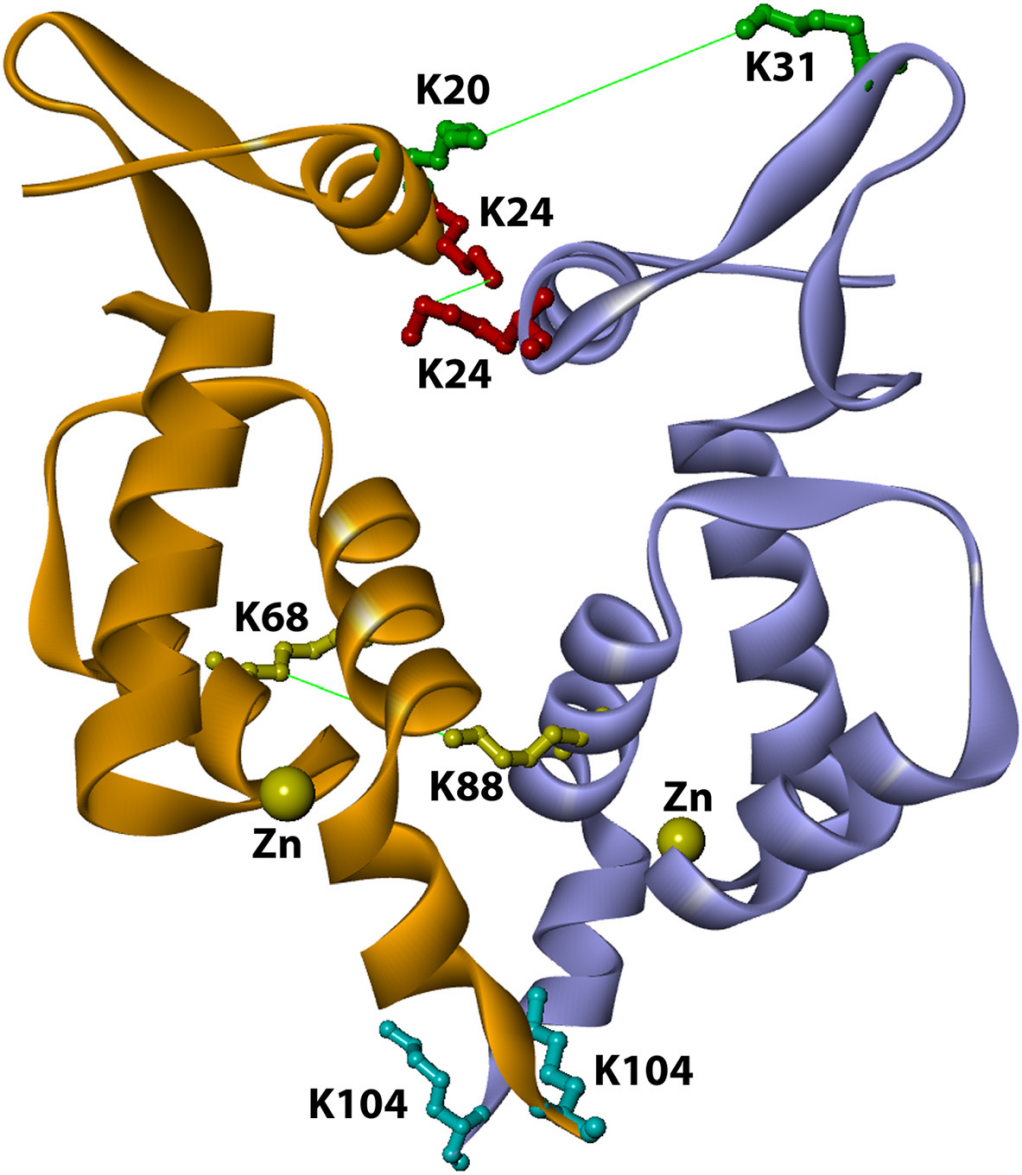
**Modeling of dimer conformation of N-terminal domain of Mo-MLV integrase.** Visualization of intermolecular crosslinking products with Swiss-PdbViewer of the dimeric interfase of NTD of Mo-MLV integrase (3NNQ pdb template, experimental crosslinking strain and 3D-Dock suite used for model). The amino acid involved in the crosslinking and its distances are indicated K31-K20 (green 20.02 Å), K24-K24 (red 15.14 Å) and K88-K68 (yellow 13.57 Å), all of them are in agreement with the spacer arm of BS^3^. K104 (light blue) and zinc atoms (yellow) are also indicated.

3D-dock suite was used to generate 10,000 possible complexes through the rotation and translation of a mobile monomer (3NNQ), around the fixed coordinates of another 3NNQ monomer. This set of possible dimers was filtered using the experimental crosslinking information, to select the complex that agrees with the distance restraint data. A distance of 21.3 Å between crosslinked lysines was used to select complex candidates. The selected dimers were submitted to an energy minimization protocol in order to optimize steric and electrostatic interactions of the residues involved in the protein - protein interface. All the dimeric structures that satisfied the distance restraints showed a parallel disposition, with the residues involved in the crosslinking distributed in the same longitudinal face of the protein and both polypeptide chains arranged in the same direction. The complex with the more stable interface, according to Multidock routine of 3D-dock suite, was chosen as the more probable NTD - NTD dimer. A ribbon representation of the selected model is presented in Figure 9, showing the lysine residues that were crosslinked. This model shows a symmetric and parallel orientation of the monomers and two points of contact. (1) In the first α-helix of the N-terminal domain (between K24-K24) and (2) the fourth α-helix of chain A (blue) with the fourth α-helix of chain B (brown) of the dimer. This parallel arrangement would be mainly stabilized by electrostatic and Van der Waals interactions, where residues: L 25, D 84, R 85 and D 92 of monomer A and K 24, L 25, D 84 and D 92 of monomer B, play the main role in complex stabilization. The complex is characterized for a ΔASA of 484 Å^2^, involving 16 residues of the first monomer and 17 residues of the second monomer. The gain in ΔG of solvation from the protein-protein interaction was estimated by PDBePISA server in 2.5 Kcal/Mol (http://www.ebi.ac.uk/msd-srv/prot_int/cgi-bin/piserver).

The solution structure of the isolated HIV-1 NTD dimer also exhibits a parallel arrangement [10], however, structural information on isolated NTDs of other integrases is not available.

It is generally thought that a tetramer is involved in the integration process. It has been proposed that this tetramer is assembled by two dimers that differ in their conformation. Differences in conformation reflect different combinations of interactions between the three domains of the enzyme. Crosslinking experiments in solution have revealed NTD-NTD interactions in the tetramer [18] that were not observed in the crystal structure of fragments of HIV-1 IN or PFV IN structure. It has been speculated that this kind of interaction may be related to a “domain swapping” phenomenon in which an interaction between the NTD and CCD domains is substituted by an interaction between the NTDs [18]. It is possible that this interaction may be evident in the isolated NTDs due to the absence of the CCD domain.

### Cysteine crosslinking

**Figure 10:**
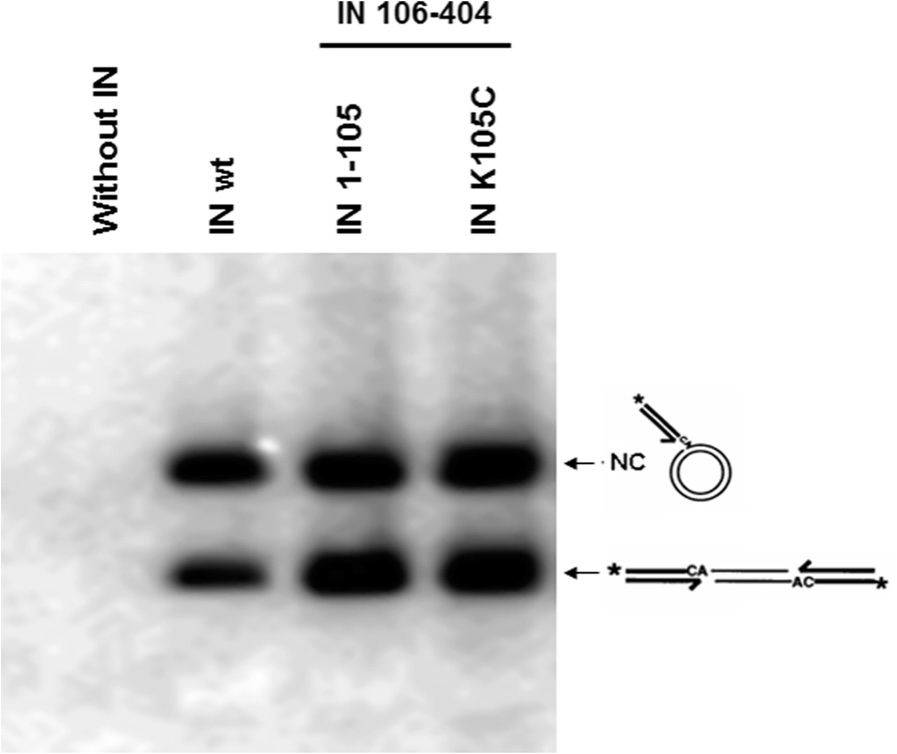
**Enzyme activity assays of wild type and IN mutants.** Integration assays were carried out with 7 pmol of wild type IN or 56 pmoles of IN 1–105 (or mutant K105C) and 7 pmoles of IN 106–404 for complementation assay. The products were electrophoresed on agarose gels. The upper product corresponds to non-concerted integration and the lower product to concerted integration. The substrate migrated off the gel.

**Figure 11:**
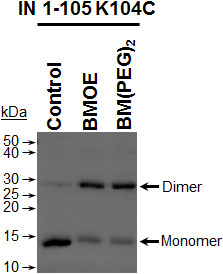
**Cysteine cross-linking.** The reaction was carried out in 50 mM Tris–HCl pH 7.0. 50 μM of IN 1–105 K104C in buffer without crosslinker (control lane) or in the presence of 1 mM of the bi-functional maleimide crosslinker BMOE or BM(PEG)_2._ The reaction was done on ice for 60 min and quenched with 10 mM DTT.

In order to test our model, we performed crosslinking studies introducing cysteine residues at position 104 in IN1-105 (K104C). This amino acid residue was chosen because the Cα of both K104 in the 3NNQ dimer is separated by 28 Å. In contrast, the distance between these residues in our model is only 10 Å. Therefore crosslinking with BMOE (8 Å) would be distinguished between both models. The mutant K104C IN1-105 was obtained by site directed mutagenesis and purified as described in Materials and Methods. This protein was able to complement a concerted integration assay that used a deletion mutant IN lacking the NTD domain (Figure 10). In addition, IN 1–105 contains two cysteine residues (C94 and 96) coordinated with zinc, that are not reactive to N-ethylmaleimide [8]. K104C IN 1–105 protein was crosslinked with BMOE and BM(PEG)_2_ of 8 and 14.7 Å in length, respectively. Figure 11 shows that both reagents produced crosslinked dimer of K104C IN 1–105 (lanes 2 and 3) indicating that the SH groups of cysteines are within 8 Å. The estimated crosslinking extent was 65% for BMOE and 70% for BM(PEG)_2._ The distance between the cysteines in the 3D coordinates of 3NNQ dimer would be more than 28 Å. Furthermore in 3NNQ both carboxyl ends are separated by interactions with helix 3 of the other monomer reducing the flexibility of the polypeptide chain at the position of K104.

In order to confirm that crosslinking was not produced by the reaction between dimers due to the high reactivity of BMOE with SH groups, the crosslinked reaction mixture was further analyzed by gel filtration on superdex S-200. After crosslinking with BMOE most of the protein eluted as a dimer (not shown).

## Conclusions

All retroviral integrases contain a HHCC zinc finger motif at the N-terminal domain that is involved in protein-protein interactions, which is essential for retroviral integration. The structure of NTD of HIV-1 IN has been solved by X-ray crystallography and NMR. However for Mo-MLV with a larger NTD domain, the only information available is the deposited x-ray structure 3NNQ. The dimeric structure represented in the asymmetric unit of 3NNQ fails to explain our crosslinking results. Studies of the isolated Mo-MLV NTD indicates that the domain behaves as a dimer in solution and as such is active in complementation of the IN activities. Therefore our aim was to determine the contacting regions of the NTD domain using chemical crosslinking and mass spectrometry to identify the dimer present in solution. Identification of intramolecular crosslinks of lysines using BS^3^ as a spacer agree with the conformation of the monomer in 3NNQ. However, the intermolecular crosslinking results did not match the interacting regions found in 3NNQ dimer.

The current approach to determine protein-protein interaction in the NTD domain of Mo-MLV integrase includes the use of the 3D coordinates of the 3NNQ monomer and experimental distance restraints obtained using lysines crosslinking and MS/MS sequencing methodologies. We propose a homodimeric model where both polypeptides are aligned in parallel with the β strands away from the interface, formed mainly by helices 1 and 4.

Our model was tested by cysteine crosslinking with BMOE and as expected a high crosslinking yield was achieved. We expect that this theoretical model could be useful to test other properties of the NTD such as its interactions with the other domains of IN and to understand the mechanisms used by this HHCC domains to regulate protein interactions in different contexts.

## Methods

### Integrase purification

*E*. *coli* BL21 (DE3) harboring the plasmid pET/IN 1–105 was used to express IN 1–105 [8]. Protein production was induced with lactose according to the protocol of Studier, F. [23]. Cells were grown at 20°C up to 5 O.D. at 600 nm in 400 mL. Cells were collected by centrifugation at 6,000 rpm for 30 min in a Sorvall RC-5, GSA rotor. The pellet was resuspended in 40 mL of lysis buffer (10 mM CHAPS, 10 mM imidazole, 300 mM NaCl, 50 mM NaH_2_PO_4_ pH 8,0 and protease inhibitor tablet (Roche) was sonicated for 6 pulses of 30 s at maximum intensity (Branson) and the cell debris was removed by centrifugation at 9,000 rpm for 40 min. 1 mL of Ni-NTA-agarose was added to the supernatant and mixed overnight by rotation in a twister VS-96 TW at 4°C. The resin was washed with 40 mL of buffer 1 (50 mM NaH_2_PO_4_ pH 8.0, 10 mM CHAPS, 1 M NaCl, 10 mM 2-mercaptoethanol, 20% glycerol for 10 min and the resin was set on a column (BioRad). The column was washed sequentially with 4 mL of: 25, 50, 100 and 250 mM imidazole dissolved in buffer 1. Fractions of 1 mL were collected. Protein was quantified by Bradford (BioRad) and analyzed by SDS-PAGE on 15% acrylamide gels.

### Construction of K104C IN 1–105 mutant

This construct was made by PCR using the plasmid pET IN1-105 as a template [8] and the mutagenic primer K104C 5’-AAA AGG ATC CTA AGA GCA GCT GGC GTT GA -3’ that included the *Bam*HI site (underlined) and the T7 promoter 5’- CTATAGTGAGTCGTATTA -3’. The 480 bp PCR product was digested with *Nde*I and *Bam*HI (NEB) and purified by electrophoresis in 1.2% agarose gels and ligated to pET 11b digested with *Nde*I and *Bam*HI. The mutation was confirmed by DNA sequencing.

### Chemical crosslinking of IN 1–105

The experimental approach to identify crosslinked lysines is based on the use of homo- bifunctional cross-linking agents that are directed primarily towards amino groups. ϵ-NH_2_ groups of lysine or the α-NH_2_ of the N-terminal amino acid of the protein would potentially react with the N-hydroxysuccinimide ester. This in turn produces intramolecular crosslinking when lysines of the same polypeptide are located at the appropriate distance. Intermolecular crosslinking can also be produced when the reactive lysines are located in different polypeptides. In our approach the reacted protein was digested with sequencing grade proteases and analyzed by MALDI TOF/TOF and LC-MS/MS to identify the position of crosslinking. For the first round analysis, the data was searched using Bioworks with +138 (for intra peptide link) and +156 as lysine variable modifications. In these studies the homobifuntional crosslinker bis(sulfosuccimidyl) suberate (BS^3^, Pierce) was used. This crosslinker spans 11.4 Å. 0.4 nmoles of the IN 1–105 (10 μM) was incubated at 25°C in 50 mM Hepes pH 7.8 and 100 mM NaCl with 100 μM of BS^3^ for 35 min. The crosslinking reaction mixture was quenched with a loading buffer of protein and 50 mM lysine pH 8.0 and subjected to SDS-PAGE and stained with Coomasie Brilliant Blue. Under these conditions 50% of IN 1–105 was crosslinked. The protein band corresponding to the crosslinked dimer was excised and digested with trypsin for mass spectrometry analysis.

### Characterization of crosslinked products of IN 1–105 by gel filtration

1.3 mg of protein in 2 mL was crosslinked as described above. The reaction products were concentrated 10-fold in an Amicon filter (10000 MWCO, Millipore) and 1.3 mg of protein (200 μL) was loaded on a Superdex S-200 column (30 × 1.7 cm Pharmacia), equilibrated with 10 mM Tris HCl pH 7.5, 0.5 M NaCl, 1 mM DTT and 5% glycerol which were connected to a diode array detector (Jasco). Fractions of 0.5 mL were collected and the protein visualized by SDS-PAGE 12% and Coomasie Brilliant Blue staining.

### Proteolytic digestion in gel

The stained gel piece was incubated with 1 mL of 50 mM of NH_4_HCO_3_ in 50% v/v acetonitrile for 1 h or until the stain disappeared. Then, the protein was reduced with 60 μL of 20 mM DTT in 50 mM NH_4_HCO_3_ for 15 min at 60°C. The solution was removed and the gel was treated with 40 mM iodoacetamide in 50 mM NH_4_HCO_3_ for 30 min at 37°C, in the dark. The gel piece was washed with 50 mM NH_4_HCO_3_ in 50% v/v acetonitrile and with 100% acetonitrile twice and dried 10 min at room temperature, then 0.8 μg of trypsin in 40 μL of 25 mM NH_4_HCO_3_, pH 7.8 were added and incubated at 37°C for 16 h. Peptides were recovered by concentration in speed vac and analyzed by LC-MS/MS (U3000 from Dionex and LTQ from Thermofisher) or MALDI-TOF/TOF (Applied biosystem).

### Proteolytic digestion in solution

The crosslinking reaction mixture (0.4 nmoles of protein) in 50 mM NH_4_HCO_3_ was treated with 20 mM iodoacetamide for 30 min at 37°C. The reaction was stopped by the addition of 20 mM DTT for 15 min. Then, trypsin in a ratio 1:20 (wt/wt) was added, and the digestion was carried out at 37°C for 16 h. The digested peptides were then analyzed by nano LC-MS/MS (U3000 from Dionex and LTQ from Thermofisher) or 4800 MALDI-TOF/TOF instrument (Applied biosystem).

### Peptide crosslinked analysis

A list of likely peptides, containing one undigested lysine for trypsin or with more than one lysine for chymotrypsin, was used as lysine modification in order to compare with unmodified peptide profile of the control without BS^3^ and search LC-MS/MS data by Bioworks software or for manual interpretation of MALDI-MS/MS data. For intra-peptide cross-linking and BS^3^ single residue modification, 138.068 and 156.0786 were added on lysine as modifications respectively. The MS/MS spectra of cross-linked peptides were manually confirmed.

### Concerted two-end integration assay

The concerted two-end integration assay is a modification of that previously described [24-26]. One pmole of the 5’-labeled substrate used for strand transfer was incubated in SST buffer (20 mM MES pH 6,4, 20 mM KCl, 5% glycerol, 10 mM DTT, 20 mM MnCl_2_ and 10% DMSO) for 30 min on ice with 7 pmol of IN wt or 56 pmoles of IN 1–105 (or mutant K105C) and 7 pmoles of IN 106–404 for complementation assay. Then 100 pmol of pUC18 DNA were added and incubated at 37°C for 1 h in a final volume of 15 μL. The reaction was stopped with 3 μL of 0.1 M EDTA, 5% SDS plus 1 μL of proteinase K (20 mg/mL) and incubated at 55°C for 1 h. The reaction products were separated by electrophoresis on 1% agarose gels. The gel was dried and exposed on a Phosphor-Imager (Bio-Rad).

### Cysteine crosslinking

Just prior to use, the IN 1–105 and K104C IN 1–105 were adjusted to 0.8 mg/mL and ZnSO_4_ (10 μM final) was added to chelate the cysteines of the zinc binding motif. The proteins were kept on ice for 30 min. The bifunctional maleimide-coupled crosslinking agent BMOE (span arm 8 Å) and BM(PEG)_2_ (span arm 14.7 Å) were prepared in DMSO just prior to use. The crosslinker was added to the protein solution at a different crosslinker/protein ratio. The reaction mixture was left on ice for 60 min and the crosslinker excess was quenched with 10 mM DTT for 30 min with further 30 min on ice. The reaction products were analyzed by SDS-PAGE and protein were stained with Coomasie Brilliant Blue.

### Monomer of N-terminal integrase

Integrase N-terminal 3D coordinates, residues 11 to 105, was a modification of 3NNQ pdb file, where the three selenomethionine residues in Chain A were edited to methionine residues.

### Dimeric complex

Integrase N-terminal dimer was built using 3D-Dock suite and experimental crosslinking restraints. In summary, one monomer of N-terminal integrase was defined to the program as a fixed structure and the other monomer as mobile. FTDock program, based on the correlation algorithm of Katchalski-Katzir plus an electrostatic function, generated 10,000 possible complexes through rotation and translation of the mobile monomer previous to the correlation calculations. Experimental cross linking information was used as a filter (filter routine in the suite) to select the complex that agreed with the distance restraint data [27]. After the model was selected, Multidock routine was used to refine the side chains of the amino acids involved in the interface and to perform a rigid body energy minimization of the complex.

## Competing interests

The authors declare that they have no competing interests.

## Authors’ contributions

DH and MA performed the work, HZ and CZ carried out the mass spectrometry analysis and sequencing. JA carried out the modeling OL, MR and DH conceived the study and coordinated the activities and writing. All authors read and approved the final manuscript and declare no conflict of interest.
